# Immunization Status against Measles, Mumps, Rubella and Varicella in a Large Population of Internationally Adopted Children Referred to Meyer Children’s University Hospital from 2009 to 2018

**DOI:** 10.3390/vaccines8010051

**Published:** 2020-01-28

**Authors:** Angela Bechini, Sara Boccalini, Cecilia Maria Alimenti, Paolo Bonanni, Luisa Galli, Elena Chiappini

**Affiliations:** 1 Department of Health Sciences, University of Florence, Viale GB Morgagni, 48–50134 Florence, Italy; cecilia.alimenti@stud.unifi.it (C.M.A.); paolo.bonanni@unifi.it (P.B.); 2 Meyer Children’s University Hospital, Viale Pieraccini 24–50139 Florence, Italy; luisa.galli@unifi.it (L.G.); elena.chiappini@unifi.it (E.C.)

**Keywords:** seroprevalence, immunological status, vaccination, internationally adopted children, measles, mumps, rubella, varicella

## Abstract

Control of vaccine preventable diseases (VPDs) is a challenge for healthcare systems. Different studies highlighted the suboptimal immunization of internationally adopted children (IAC). To evaluate the immunization status against measles, mumps, rubella (MMR), and varicella (V) in a large cohort of IAC, data at first screening visit of all IAC (<18 years) consecutively referred to Meyer Children’s University Hospital (Florence, Italy) from 2009 to 2018 were collected and analyzed. In total, 1927 children (median age: 5.99 years, interquartile range: 3.33–8.21) were enrolled. More than half of IAC were unprotected against MMR-V. The reliability of the vaccination documentation of the country of origin was poor, since more than a quarter of the IAC serologically tested were not protected against MMR-V, despite the vaccination documentation attesting previous vaccination. This was significantly more pronounced in children aged 15–18 years and in those originating from Africa. High rate of discordant serological results/documentation brings up questions regarding the optimal management of IACs, and suggests a rapid, careful, and complete assessment of immunization status timely after IAC’s arrival. Serological testing of IAC of all ages followed by vaccination of seronegative children should be provided.

## 1. Introduction

The control of vaccine preventable diseases (VPDs), such as measles or varicella, is a priority for healthcare systems. Outbreaks have been reported worldwide in recent years due to insufficient immunization coverage among the general population [1]. Accordingly, recent epidemiological data on VPDs in the European Union and European Economic Area (EU/EEA) demonstrate ongoing transmission in the context of insufficient vaccine coverage [1].

For instance, measles continues to be a threat in EU/EEA countries. From 1 January 2019 to 14 July 2019, 10,958 cases were reported in adults and children by the European Centre for Disease Prevention and Control [2,3]. The highest numbers were described in France, Bulgaria, Italy, Poland, and Lithuania [4]. In fact, Italy never reached the elimination status (interruption for at least 36 months). There were 1605 cases of measles and 19 cases of rubella reported in Italy during 2019 from the 1 January to the 31 October [5].

Various studies highlighted the need to maintain protective antibody titers levels in order to reach the thresholds of herd immunity for those vaccines for which herd immunity is possible, such as measles, mumps, rubella, and varicella (MMR-V) [1,6]. The threshold to be reached and maintained to ensure the herd immunity effect for MMR and V is 95% of vaccination coverage for 2 doses as established by the Italian National Immunization Programme (NIP) [7].

The Italian NIP is one of the most complete worldwide. However, in 2018, the vaccination coverage at 24 months of age for measles, mumps, and rubella was 93% and for varicella was 74% [8]. As a matter of fact, the herd immunity threshold for varicella in Italy was estimated around 70% according to Nardone et al [9].

Only in 2017 varicella’s vaccine was introduced nationally [7]. The universal varicella vaccination was introduced progressively in some regions since 2003 [10].

Nationally, varicella’s vaccination was recommended starting from 2017. In Tuscany, it was introduced since 2008 showing reduction of notification and hospitalization for varicella with a varicella coverage of 76–84% from 2010–2012 [10,11,12].

In 2018, 1394 children were internationally adopted in Italy. Italy is the second nation at a global level with the highest rate of international adoptions behind the United States of America [13].

The Italian NIP recommends updating of the vaccination of migrants, including internationally adopted children (IAC), as soon as possible after their arrival, in order to prevent the spread of VPDs. In fact, a large proportion of IAC are likely to be susceptible to VPDs because of suboptimal immunization [14,15]: Children may be unvaccinated, partially vaccinated, or their immune status may be uncertain or unknown [16]. Moreover, their native country and adoptive country may have different immunization policies and schedules [17,18,19,20].

Proper documentation of immunization is a worldwide problem for IAC and also for migrants and asylum seekers [21].

Few studies are available reporting the percentage of IAC with protective antibody titers for VPDs. Protective antibody titers were reported in 12–95% of IAC for measles, in 61–94% for rubella, and in 48–79% for mumps [14,19,20,22,23,24,25,26,27]. Antibody levels for varicella varied from 30–100% [14,19,20,24,25,26,27]. Updated information regarding the vaccination status of IAC is of paramount importance in order to support decisions regarding vaccination programs soon after arrival in the adoption country.

Therefore, the aim of the current study was to analyze measles-mumps-rubella-varicella (MMRV) vaccination documentation available and immunological status in a large population of IAC referred to a single center in Tuscany (Italy) over a 10-year period.

## 2. Materials and Methods

### 2.1. Study Population

Between January 2009 and December 2018, we enrolled all the IAC consecutively referred to the Meyer Children’s University Hospital by their adoptive parents, who consented to the screening evaluation. These children were assessed during the first evaluation, following the protocol recommended by the Italian national working group on migrant children (GLNIB) [19], as previously described [19] in particular focusing on immunization status.

Italian adopted children were excluded from the study. Conversely, IAC from all over the world were included if they were under 18 years of age and who had at least one serological test among measles, mumps, rubella, and varicella performed at the first evaluation. In addition, IAC whose parents did not sign consent were excluded from the study. 

The study received approval by Meyer Children’s University Hospital Ethics Committee, the Ethical Code Number is 15/2010. Parents who consented to their adoptive children participating in the study have signed a written informed consent. 

### 2.2. Study Design

This is a retrospective monocentric study. For each child included in the study, the following information was collected and entered in an electronic database, following a standard operative protocol for IAC developed internationally and adopted at Meyer Children’s University Hospital [19,26,28]. The electronic database was a secured system to protect patient information. Briefly, the following information was retrieved for the purpose of the present study: Country of origin, gender, age at first observation, vaccine documentation for MMRV, results of serological tests for measles, mumps, rubella, and varicella. At the first evaluation, all the children underwent a venepuncture and laboratory assessment including serologic tests. Staff involved for the venepuncture and laboratory assessment were appropriately trained to do so.

All the other laboratory examinations were performed in the same laboratory at the Meyer Children’s University Hospital, using standardized techniques and according to manufacturers’ instructions.

In particular, serology tests for measles, rubella, varicella, and mumps were performed using a chemiluminescent immunoassay (CLIA) technology (LIAISONXL System, DiaSorin, Saluggia (VC), Italy) with a definition of seropositive samples when their antibody titers were >16.5 mIU/mL for measles, >15 IU/mL for rubella, >165 mIU/mL for varicella, and >11AU/mL for mumps.

### 2.3. Seroprevalence of Antibody Protection against Measles, Mumps, Rubella, and Varicella

Children were subdivided according to age in five groups (<1y, 1–4 y, 5–9 y, 10–14 y, 15–18y) and classified as protected (seropositive) or unprotected (seronegative) against each specific VPD on the basis of serotesting results. Antibody seroprevalence for each age group was calculated and expressed as a percentage.

### 2.4. Concordance between Vaccine Documentation and Serotesting Results

Vaccine documentation was assessed, considering the number of doses recorded and the concordance between the available documentation and serotesting results for measles, mumps, rubella, and varicella. These data were recorded and entered in tables.

### 2.5. Statistical Analysis

Data were reported as median and interquartile range (IQR) or absolute numbers and percentages. Fisher exact test and Chi-square test were used to compare categorical variables, as appropriate. All statistical analyses were carried out using the SPSS (Statistical Package of Social Sciences, Chicago, IL) for Windows software program version 19.0. A *p* value < 0.05 was considered significant. 

## 3. Results

### 3.1. Characteristics of the Study Population 

In the decade 2009–2018, 2200 IAC from 64 countries were assessed for post-adoption screening. Of this initial group, 1927 were eligible for our study, while 273 were excluded from the evaluation because at least one serological test result was not available. 

Considering serology results and/or documentation available, 1870 IAC were assessed for measles, 1868 for rubella, 631 for varicella, and 844 for mumps. Thus, 96.9% and 97% of the study population were included in the study for rubella and measles, respectively. Of the study population, 43.8% was included for mumps, whereas only 32.7% of the population was included in the study for varicella immunization status. The median age at first evaluation was 5.99 (IQR: 3.33–8.21) years, 40.1% of the children were girls (773/1927) (Table 1).

Forty percent of the IAC were adopted from Europe (777/1927), most of them were from Russia; 21.7% from Central or South America (419/1927); 19.9% were adopted from Asia (384/1927); 18% from Africa (347/1927) (Table 1). The most represented countries were Russia (458/1927, 23.8%), Colombia (146/1927, 7.6%), India (144/1927, 7.5%), and Ethiopia (125/1927, 6%). Since the most populated area of Russia is in Europe (and not in Asia), the data from Russia were entered in Europe.

Younger children came from Asia while the older were born in Americas (Table 1). Children aged 1 year or younger accounted for 2.6% of IAC, children between 1 and 4 years accounted for 39.2% of IAC, and children aged between 5 and 9 years accounted for 47.5% of IAC. Only 9.3% of the IAC were 10 to 14 years old, and older IAC belonging to the age group 15–18 years accounted for 1.3% of the population. IAC aged between 5 and 9 years represented the largest age group (Table 2).

### 3.2. Evaluation of the Immunological Coverage by Means of Serological Tests

Most of the children enrolled by Meyer Children’s University Hospital during the study period (2009–2018) were tested to evaluate the protective antibodies titers against measles, rubella, varicella, and mumps. Protective antibody titers were recorded in 64.9% of IAC for measles, in 60% for mumps, in 67.9% for rubella, and in 54% for varicella. Moreover, Africa was the continent with the highest percentage of unprotected children for measles (*p* < 0.0001), rubella (*p* < 0.0001), while IAC from Asia showed the highest percentage with unprotective antibodies for varicella (*p* < 0.0001) (Table 3).

**Figure 1:**
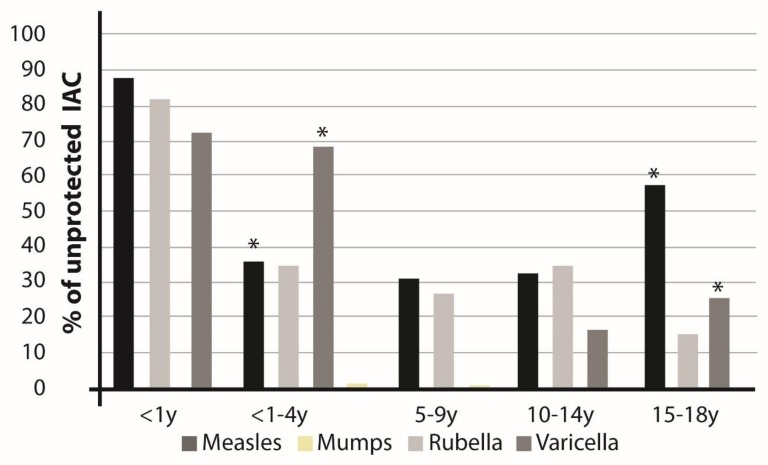
Percentage of internationally adopted children (IAC) with unprotective antibody titers (seronegative) by age groups.

The large majority of children <1 year of age were unprotected against the four VPDs investigated. Moreover, 57.7% of older children were unprotected for measles (* *p* < 0.001 for measles and varicella in 1–4 y vs. 15–18 y). In the age group 1–4 years, 36% of IAC were unprotected for measles, 35% for rubella, and 69% for varicella (Figure 1).

From ages 5 and up to 14 years, we observed the highest percentage of protected IAC.Notably, almost 60% of children aged 15–18 years were unprotected for measles.

Documentation of the vaccine received in the country of origin was not always available (Table 4). In particular, a discrepancy between documentation indicating previous vaccinations and unprotective (seronegative) antibody titers was evidenced in 25.6% of the children for measles, 24.9% for rubella, 53.3% for varicella, and 25% for mumps (Table 4).

By evaluating the serological results according to documentation records and age groups, it is evident that under 1 year of age there was a high percentage of IAC without serological protection: 87.75% for measles, 82.35% for rubella, and 72.73% for varicella (Figure 1).

The discrepancy between serological results and vaccine documentation was reported in Africa from 49% to 54% depending on the disease considered. For Americas, the discrepancy varied from 47% to 70%. The discrepancy varied for Asia from 35% to 42% and for Europe from 33% to 58% (Table 5).

Considering age groups, discrepancies were observed from 12% to 27% for IAC <1 year of age, from 33% to 38% for IAC from 1 to 4 years of age, from 42% to 64% for IAC from 5 to 9 years of age, from 32% to 78% for IAC from 10 to 14 years of age, and for adolescences 15–18 years of age from 50% to 73%.

According to documentation of vaccinations, except for mumps vaccination records, most children did not receive any dose of vaccine (Table 6). Most IAC <1 year of age had no documentation of vaccination. More often IAC from 1–4 and from 5–9 age groups had some documentation of previous vaccination. Particularly, children in the 5–9 age group were those who had more frequently recorded the second dose.

## 4. Discussion

In the current study we analyzed the measles-mumps-rubella-varicella (MMRV) vaccination status and serological data available in a large population of IAC referred to a single center in Tuscany (Italy) over a 10-year period. To our knowledge, this is one of the most numerous studies including data from more than 1900 IAC collected over a 10-year period. A large proportion of children resulted seronegative toward measles (35.1%), rubella (32.1%), varicella (45.9%), and mumps (40%). These figures are more pronounced considering African children for measles and rubella (46.3% and 41.2%, respectively) and Asian children for varicella (60%). As expected, children <1 year of age were more commonly unprotected (72–88%), as the first dose of MMR-V vaccine is recommended between 12 and 15 months of age. The second dose is recommended during childhood. The minimum interval between first dose and second dose is 4 weeks. In Italy the second dose is recommended at 5–6 years of age. Age group 1–4 years may have only received 1 dose and would not be fully protected. Our results show that in the age group 1–4 years, a percentage between 35–69% of children were unprotected (Figure 1) particularly for measles, rubella, and varicella. From ages 5 and up to 14 years, we observed the highest percentage of protected IAC. Notably, almost 60% of children aged 15–18 years were unprotected for measles, representing an age group which should be considered particularly at high risk. It shouldn’t be taken for granted that adolescents have already received vaccination against MMRV or have already acquired the infection.

Several studies assessing the proportion of children (IAC and migrants) with protective antibody titers against different VPDs are available. These studies reported a high variability of antibody protection rates. In particular, protective antibody titers were reported in a range of 12–95% for measles, 61–94% for rubella, 48–79% for mumps, and 30–100% for varicella. Our data/results were in accordance with the results of previous studies [6,11,14,15,16,19,20,21,22,23,24,25,26,27,28,29,30].

The written documentation of the IAC may not accurately reflect the immunization status [24,29]. 

The lack of vaccine documentation reliability has been reported in literature and numerous studies have correlated this unreliability with no protection or under-immunization [14,20,22,30,31,32]. Our data underline the poor reliability of vaccine documentation, when available: Discrepancies between serological test results and vaccine documentation were observed in about one-quarter of IAC for MMR and more than half the children for varicella.

These studies report a variable documentation reliability in the range of 62–83% for measles, 38–86% for rubella, 55–75% for mumps, and 47–91% for varicella. In particular, in the most recent study conducted by Giordano et al., the reliability of the documentation for measles, mumps, rubella, and hepatitis B were assessed [14]. This study showed a concordance between protective antibody titers and written documentation stating the vaccinations which had been carried out in the country of origin. The concordance was 78% for measles, 65.2% for mumps, 83.6% for rubella, and 71.2% for hepatitis B [14].

According to the literature, IAC’s place of birth has been described to be related with the reliability of immunization records. For example, African and Central American IAC had a lack of vaccine documentation compared with Eastern European and Asian IAC [32]. Some authors have reported that certificates were written with the same pencil, reported in the same date of consecutive months, or even dated before the child’s birth [22]. Some other obstacles identified when reviewing the immunization records were translation issues and misinterpretation of written reported brand name of the vaccine.

Any vaccines the children may have received could be ineffective due to suboptimal storage and transport or because they were administered after the expiration date [16,17]. IAC are often malnourished and have severe co-infections or immunodeficiencies and these increase their risk of immunization failure [19,20].

Despite the existence of vaccination records, these can be unreliable and justify the importance of serology test promptly after the arrival of IAC.

Our study has several limitations. During the 10-year study period some investigations included in the screening protocol changed, and some tests were not performed in the whole population: In fact, the large majority of the children referred to Meyer Children’s University Hospital and included in the study were evaluated as per protocol, but for some variables some data were missing, possibly due to the physicians’ incomplete adherence to the screening protocol or to an incomplete collection of data in medical records. Indeed, it must be considered that our center was able to recruit only a part of all the adopted children arriving in Tuscany, as some parents may have consulted their primary care pediatrician or no one at all. Lastly, there is no absolute association between being unprotected and not having protective antibody titers, since immune response to vaccines is complex and relies on different immunologic mechanisms, which are not all explored by serology testing [33].

## 5. Conclusions

Our data underline the importance of a rapid, careful, and complete assessment of immunization status in IAC. Many factors, such as lack of public health infrastructure or maintenance of the cold chain for the storage of the vaccines or malnutrition especially in low- and middle-income countries, influence the serological pattern of these children. This study highlighted that more than half of the newly arrived IAC were unprotected against MMRV. Notably, even if vaccination documentation of the country of origin was available, this was not always reliable: a quarter of the IAC were not protected in the current study. Our data suggest that vaccination status and serological assessment should be promptly performed on all IAC after their arrival in order to prevent outbreaks of VPDs.

## Figures and Tables

**Table 1:** Characteristics of the study population by continent of origin, gender, and median age.

	Africa	Americas	Asia	Europe	Total
Population (*n*)	347 (18%)	419 (21.7%)	384 (19.9%)	777 (40.3%)	1927 (100%)
Median age (y)	5.19	7.37	4.81	6.19	5.99
IQR	2.66–6.79	5.30–9.34	2.01–7.06	3.73–8.26	3.33–8.21
Females (*n*)	145 (41.8%)	193 (46.1%)	175 (45.6%)	260 (33.5%)	773 (40.1%)
Males (*n*)	202 (58.2%)	226 (53.9%)	209 (54.4%)	517 (66.5%)	1154 (59.9%)

**Table 2:** Continent of origin of the internationally adopted children (IAC) referred to Meyer Children’s University Hospital, Florence, in the period (2009–2018), by age group (%).

Age Group Year	Africa % (*n* = 347)	Americas % (*n* = 419)	Asia % (*n* = 384)	Europe % (*n* = 777)	Total % (*n* = 1927)
<1	6.3	0.2	6.3	0.5	2.6
1–4	47.6	21	48.2	40.8	39.2
5–9	38.6	62.8	41.1	46.3	47.5
10–14	5.5	14.6	3.6	11.1	9.3
15–18	2	1.4	0.8	1.3	1.3
total	18%	21.7%	19.9%	40.3%	100%

**Table 3:** Percentage of children in the study with unprotective antibody titers (seronegative) against measles, rubella, varicella, and mumps, by continent of origin.

	UNPROTECTED/Total Number of Children for Each Disease	Africa (*n* = 347)	Americas (*n* = 419)	Asia (*n* = 384)	Europe (*n* = 777)
Measles n/N (%)	657/1870 (35.1%)	156/337(46.3%)	147/409 (35.9%)	159/377 (42.2%)	195/747 (26.1%)
Rubella n/N (%)	600/1868 (32.1%)	139/337 (41.3%)	89/408 (21.8%)	136/373 (36.5%)	236/750 (31.5%)
Varicella n/N (%)	290/631 (46.0%)	63/120 (52.5%)	42/134 (31.3%)	102/170 (60.0%)	83/207 (40.1%)
Mumps n/N (%)	10/25 (40.0%)	2/15 (13.3%)	0/193 (0.0%)	5/122 (4.1%)	3/514 (0.6%)

**Table 4:** Comparison between documentation recorded and serological tests performed in Italy.

VPD	Immunization Status According to Documentation *n* (%)	Immunization Status According to Serological Test *n* (%)		Total	
		Protected	Unprotected		
Measles	Recorded	717 (74.4)	247 (25.6)	964	*p* < 0.001
	Not recorded	496 (54.7)	410 (45.3)	906	
Rubella	Recorded	674 (75.1)	223 (24.9)	897	*p* < 0.001
	Not recorded	594 (61.2)	377 (38.8)	971	
Varicella	Recorded	35 (46.7)	40 (53.3)	75	*p* = 0.172
	Not Recorded	306 (55.0)	250 (45.0)	556	
Mumps	Recorded	6 (75.0)	2 (25.0)	8	*p* = 0.294
	Not Recorded	9 (52.9)	8 (47.1)	17	

**Table 5:** Number and percentage of IAC with concordance and discordance between documentation records and serological results by continent of origin and age group.

Continent	Concordance/Discordance	Measles		Rubella		Varicella	
Africa	Concordance	168	49.85%	154	45.70%	61	50.83%
	Discordance	169	50.15%	183	54.30%	59	49.17%
Total		337		337		120	
Americas	Concordance	214	52.32%	212	51.96%	40	29.85%
	Discordance	195	47.68%	196	48.04%	94	70.15%
Total		409		408		134	
Asia	Concordance	245	64.99%	234	62.73%	97	57.06%
	Discordance	132	35.01%	139	37.27%	73	42.94%
Total		377		373		170	
Europe	Concordance	500	66.93%	451	60.13%	87	42.03%
	Discordance	247	33.07%	299	39.87%	120	57.97%
Total		747		750		207	
Age group							
<1 yrs	Concordance	43	87.76%	41	80.39%	8	72.73%
	Discordance	6	12.24%	10	19.61%	3	27.27%
Total		49		51		11	
1–4 yrs	Concordance	455	62.24%	473	64.97%	143	67.14%
	Discordance	276	37.76%	255	35.03%	70	32.86%
Total		731		728		213	
5–9 yrs	Concordance	514	58.08%	444	50.17%	111	36.27%
	Discordance	371	41.92%	441	49.83%	195	63.73%
Total		885		885		306	
10–14 yrs	Concordance	102	56.98%	88	49.44%	17	21.79%
	Discordance	57	31.84%	90	50.56%	61	78.21%
Total		179		178		78	
15–18 yrs	Concordance	13	50.00%	5	19.23%	6	26.09%
	Discordance	13	50.00%	21	80.77%	17	73.91%
Total		26		26		23	

Note: Concordance = seropositive IAC with documentation of previous vaccination and seronegative with unrecorded vaccination. Discordance = seropositive IAC despite the absence of vaccination records and seronegative while the documentation attests the vaccination as received.

**Table 6:** Number of doses documented for IAC by age groups.

	Age Group	0 Dose	1 Dose	2 Doses
Measles (*n* = 1870)	<1 y	45	4	0
	1–4 y	368	318	45
	5–9 y	446	246	193
	10–14 y	93	33	53
	15–18 y	20	3	3
Rubella (*n* = 1868)	<1 y	50	1	0
	1–4 y	407	296	25
	5–9 y	455	258	172
	10–14 y	102	32	44
	15–18 y	23	2	1
Varicella (*n* = 631)	<1 y	11	0	0
	1–4 y	186	23	4
	5–9 y	273	25	8
	10–14 y	75	2	1
	15–18 y	23	0	0
Mumps (*n* = 844)	<1 y	3	0	0
	1–4 y	6	286	23
	5–9 y	7	258	178
	10–14 y	1	33	45
	15–18 y	0	3	1
